# Atrophy of gluteus maximus among women with a history of chronic low back pain

**DOI:** 10.1371/journal.pone.0177008

**Published:** 2017-07-17

**Authors:** Amy H. Amabile, John H. Bolte, Saskia D. Richter

**Affiliations:** 1 Department of Physical Therapy, Thomas Jefferson University, Philadelphia, Pennsylvania, United States of America; 2 School of Health and Rehabilitation Sciences, The Ohio State University, Columbus, Ohio, United States of America; 3 Department of Biomedical Education and Anatomy, The Ohio State University, Columbus, Ohio, United States of America; Semmelweis Egyetem, HUNGARY

## Abstract

**Background:**

Although the relationship between low back pain (LBP) and the size of certain trunk muscles has been extensively studied, the relationship between gluteus maximus (GM) size and LBP has been only minimally examined. Determining whether such a relationship exists would help improve our understanding of the etiology of LBP, and possibly provide a rationale for the use of therapeutic exercise interventions targeting GM with LBP patients. The objective of this study was to compare gluteus maximus cross-sectional area in individuals with chronic LBP, and in a group of individuals without LBP. Our hypothesis was that individuals with LBP would have greater atrophy in their gluteus maximus muscles than our control group.

**Materials and methods:**

For this case-control study, we analyzed medical history and pelvic computed tomography (CT) scans for 36 female patients with a history of chronic LBP, and 32 female patients without a history of LBP. Muscle cross-sectional area of gluteus maximus was measured from axial CT scans using OsiriX MD software, then was normalized to patient height, and used to compare the two groups. The number of back pain-related medical visits was also correlated with gluteus maximus cross-sectional area.

**Results:**

Mean normalized cross-sectional area was significantly smaller in the LBP group than in the control group, with *t* = 2.439 and *P*<0.05. The number of back pain-related visits was found to be significantly correlated with normalized cross-sectional area, with *r* = -0.270 and *P*<0.05.

The atrophy seen in the present research may reflect incidental disuse atrophy seen with LBP, which is present in many muscle groups after prolonged immobilization or with a sedentary lifestyle.

**Conclusions:**

This research demonstrated a previously only minimally explored relationship between gluteus maximus cross-sectional area and LBP in women. Further research is indicated in individuals with varying age, sex, and LBP diagnoses.

## Introduction

Low back pain (LBP) is a common and often debilitating health problem in the United States and worldwide. According to the 2012 National Health Interview Survey, 27% of all Americans over the age of 18 had experienced LBP in the prior three months, with the highest incidence (32%) among 45 to 64 year olds [1]. Worldwide prevalence of LBP has been estimated at 38% annually for all age groups [2].

LBP is a major cause of workplace disability, absenteeism, and expense, and its impact is felt among workers throughout the private sector and at all levels and branches of the military [2–7]. LBP was found to be the second most common cause of lost work time, and led to the highest rate of absenteeism, among 28,902 randomly sampled workers from a variety of industries [7]. LBP-related conditions are also responsible for the highest five-year cumulative risk of disability among active duty US Army personnel [5].

Deficits in trunk and hip muscle strength [8,9], endurance [10,11], and motor control [12–14] have been identified in persons with LBP, yet it is unknown if these deficits are a cause or an effect of LBP. Although multifidus (MF) and other trunk muscles have been extensively studied with regard to LBP [15–19], the possible role of gluteus maximus (GM) in the genesis of LBP has been only minimally examined. GM has a well-established role in the lifting of loads from a fully flexed position [20–22] and, in certain conditions, lifting has been identified as a cause of LBP [6,23–25]. GM is most important during the first 50% of trunk extension from full flexion, when intervertebral discs (IVDs) are known to be most at risk for herniation [22,26]. Thus, weak GM may lead to an improper lifting technique not just when IVDs are most vulnerable to herniation, but also at the point when maximal stress is placed on the spinal ligaments. Although IVD herniation clearly does not always lead to LBP, the possible relationship of weak GM to increased herniation risk is worth consideration as a factor in LBP genesis. There is also evidence that GM compensates for the erector spinae (ES) muscles by becoming more active when the ES become fatigued [27], which could help protect against certain low back injuries.

In a previous study [28], we found that GM cross-sectional area (CSA) correlated significantly with MF CSA in a cadaver sample of adult males. As correlations between two variables are not guaranteed to be transitive to a third variable, such as LBP in this case [29], we were motivated to further investigate the direct relationship of GM atrophy to LBP. The purpose of the present study was to compare GM CSA in individuals with chronic LBP with GM CSA in a control group of individuals without chronic LBP. Our hypothesis was that patients with chronic LBP would have greater atrophy in their GM muscles than patients without LBP.

## Materials and methods

### Sample description

Our sample consisted of 68 women 40–69 years of age, 36 of whom had a history of chronic LBP, and 32 without any known history of LBP. This was a convenience sample of patients identified through the Ohio State University (OSU) Honest Broker Protocol, which is described below. Female patients were chosen to control for the known variance of muscle CSA between men and women [30–32], and because pelvic computed tomography (CT) scans of women are widely available. Pelvic CT scans were used in this study because the gluteal region is well-visualized in such a scan. All patients had received a minimum of one year of medical care as inpatients or outpatients within the OSU Wexner Medical Center care system. All patients in the experimental group had at least two back pain-related medical visits, with a history of LBP lasting at least three months, in accordance with the most commonly used definition of chronic LBP [15,33–36]. This also follows, in part, the recommendations of the National Institutes of Health Research Task Force on chronic LBP, which defines chronicity as comprising a three-month history of LBP and pain on at least half of the days in the prior six months [37].

Exclusion criteria included any history of diagnoses that can cause LBP or muscle atrophy, including: benign or malignant neoplasm, central nervous system disorders, other chronic pain syndromes, connective tissue or rheumatoid disorders, upper urinary tract disorders, reproductive organ dysfunction, and HIV/AIDS. The control group was subject to the same exclusion criteria as the experimental group, with the additional exclusion criteria of an absence of any documented history of LBP.

Patients were identified through the OSU Center for Clinical and Translational Science, Honest Broker Protocol. This protocol allows access to de-identified patient data from the OSU Wexner Medical Center electronic medical record (EMR) system, and as such is exempt from Institutional Review Board approval. In order to obtain a set of patients for whom imaging was available for the GM muscle, patients were selected from a master list of all female patients at OSU, aged 40–69, who underwent a pelvic CT scan in 2013 or 2014. This age range, which encompasses the cohort with the most cases of chronic LBP in the United States [1], was chosen in order to limit the effects of age on outcomes, since both LBP and muscle CSA are known to vary with age [9,38–40]. Patient height was also obtained from the patient’s EMR for normalization of CSA measurements. History of chronic LBP was assessed based on the presence of a “reason for visit” of “back pain” or “lower back pain” in the patient’s EMR. Because over 60 different International Classification of Diseases (ICD-9) codes have been identified that pertain to back pain [41], we chose to base our definition of chronic LBP on the patient’s reported reason for visit rather than the ICD-9 code. Table 1 lists mean patient characteristics for the two groups, and ICD-9 codes for our chronic LBP sample are listed in Table 2.

**Table 1:** Descriptive characteristics of study sample.

Variables	Low Back Pain Patients (n = 36)		Control Patients (n = 32)		*P-value
	Mean (SD)	Range	Mean (SD)	Range	
Age (years)	51.6 (8.7)	40.0–67.0	51.3 (8.6)	40.0–67.0	0.866
Height (cm)	165.47 (4.66)	157.48–175.26	165.95 (5.51)	154.94–175.26	0.699
Number of Back Pain Visits	4.5 (3.0)	2–14	0.0 (0.0)	n/a	**0.000**

* Student's t-test.

Statistical Significance (*P* < .05).

**Table 2:** ICD-9 codes for chronic low back pain sample.

ICD-9 Codes	Number of Patients
Lumbago (724.2)	18
Backache Unspecified (724.5)	7
Sciatica (724.3)	4
Thoracic or Lumbosacral Neuritis or Radiculitus (724.4)	3
Non Allopathic Lesions of Lumbar Region (739.3)	2
Post Laminectomy Syndrome of Lumbar Region (722.83)	1
Lumbar Disc Displacement without Myelopathy (722.1)	1

ICD-9 = International Classification of Diseases

**Fig 1:**
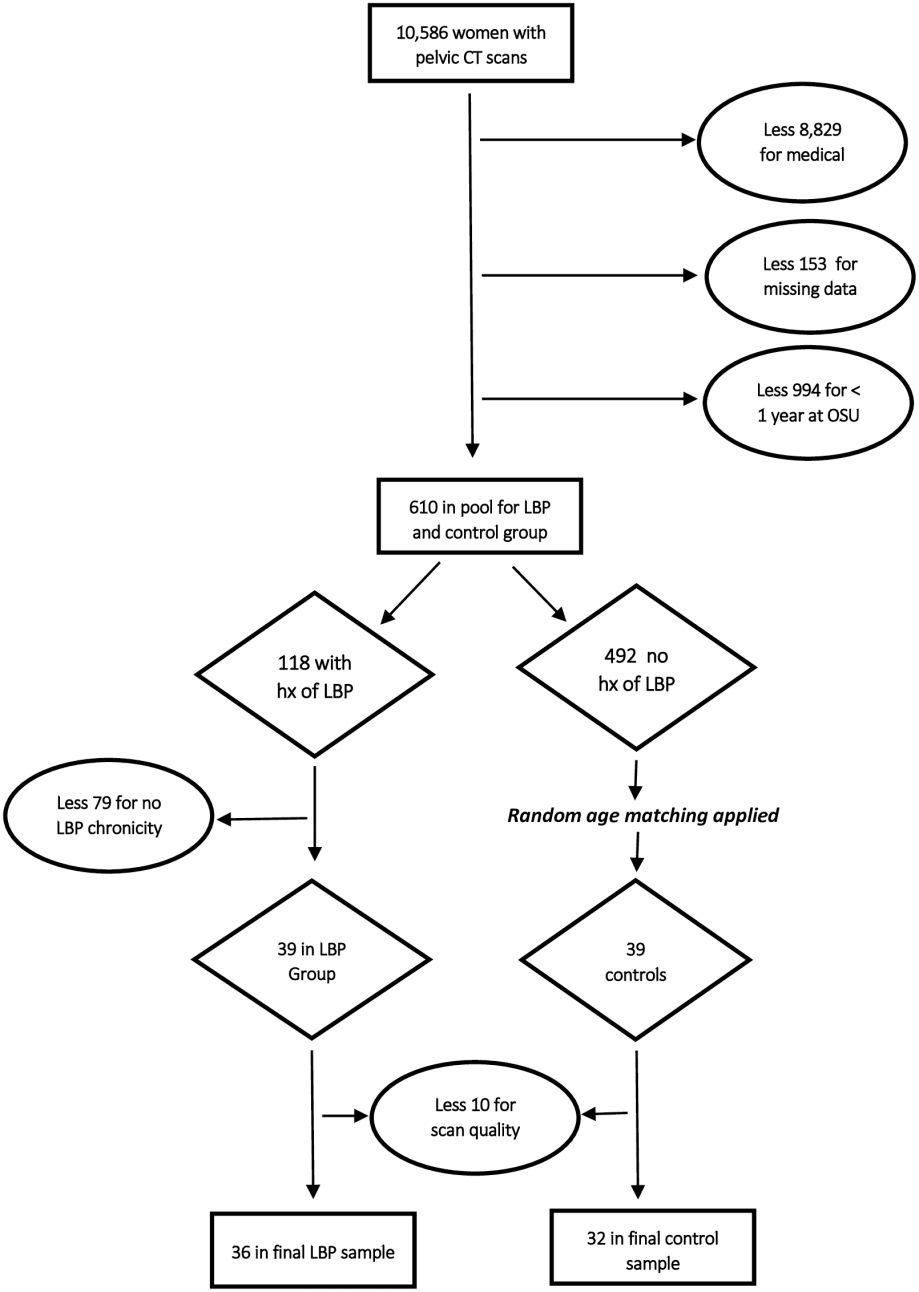
Sampling methodology.

Fig 1 illustrates our sampling methodology. Our initial search identified 10,586 women in the selected age range who had undergone pelvic CT scans at OSU. After applying medical exclusionary criteria and eliminating patients with missing data fields, or with less than one year of care at OSU, a pool of 610 women remained, of whom 118 had a history of LBP. Of these, 39 had greater than one visit for back pain with episodes lasting at least three months. Thirty-nine age-matched controls were then selected randomly, using a random number generator function in Excel 2013. After elimination due to poor scan quality, 36 experimental and 32 control patients remained (Table 1).

### Measurements and procedures

Patients underwent CT scanning using a Somatom Definition (Siemens, Erlangen, Germany), Somatom Flash (Siemens, Erlangen, Germany), Lightspeed VCT (GE Healthcare, Waukesha, Wisconsin) or Brightspeed (GE Healthcare, Waukesha, Wisconsin) CT scanner at 100 to 130kV. Slice thickness was 5.0 millimeters.

**Fig 2:**
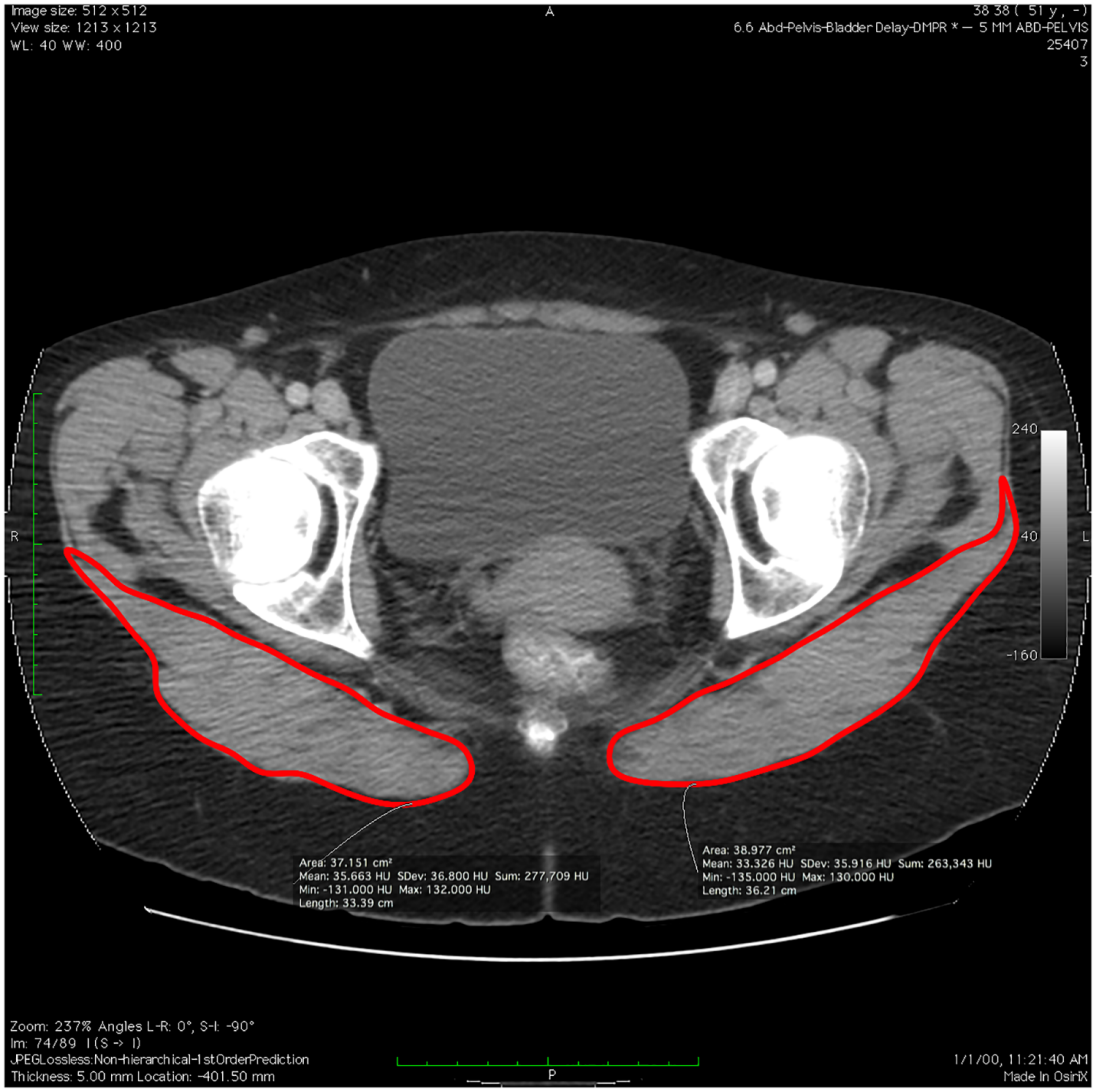
Measurement of gluteus maximus cross-sectional area.

Cross-sectional area measurements were performed using OsiriX MD software (version 7.0.2, Pixmeo, Geneva, Switzerland) software on an iMac computer (Apple Computer, Cupertino, California). Each muscle CSA was determined by using the OsiriX region of interest pencil tool and was manually traced by the study’s principal author (Fig 2). We used axial slices at the thickest point of the muscle, in order to conform to the common definition of anatomical CSA [42]. Based on a preliminary analysis of patient scans from the present sample, the thickest point was determined to be at the level of the apex of the coccyx.

Each CSA was measured three times in one session, and then an average of these measurements was taken, based on the protocol used by McGill et al [43] to maximize measurement reliability. Although CSA was measured by a single rater, an interrater study of GM CSA measurements was performed on a subset of 28 patients from our sample, with one rater blinded to the medical histories of the patients. The results of this study showed excellent reliability between the measurements of the two raters, with an intra-class correlation coefficient (ICC) of 0.938.

### Statistical analysis

Data were analyzed using SPSS version 22 (Armonk, NY) for Windows. CSA was measured in centimeters squared, and was normalized to stature by taking the ratio of CSA to the square of patient height in centimeters, based on Heymsfield et al’s [44] findings vis-a-vis the scaling of lean muscle to stature. Intraclass correlation coefficients were derived to test the reliability of the CSA measurements. Normality tests showed that some data were not normally distributed, and thus both parametric and non-parametric tests were performed when appropriate and so noted. A Wilcoxon signed-rank test was used to compare the GM CSA on the right and left sides. Mean normalized GM CSAs were compared using Student’s *t* test, and Pearson product-moment correlations were performed to show the relationship between number of back pain-related visits and GM CSA. Statistical significance was defined as *P*<0.05.

## Results

Characteristics of the chronic LBP and control patients are shown in Table 1, showing no significant differences in age or height between the groups. A Wilcoxon signed-rank test showed that there was no statistically significant difference between the GM CSAs of the right and left sides in our patients. Although certain studies [15,16,18,19] of unilateral LBP have attributed muscle asymmetries to ipsilateral LBP, other researchers [45–47] have found that paired muscles are not always symmetrical in a normal population. The desire to capture bilateral changes that may occur due to LBP, along with the lack of availability of sidedness information in our data set, led us to thus combine the two sides for measurement.

The GM CSAs in the chronic LBP and control samples were found to be normally distributed. We then used Student’s *t* test to compare mean normalized GM CSAs, and found a significantly smaller CSA for the chronic LBP group than for the control group, (*t*(66) = 2.439; *P*<0.05; Table 3). A Pearson product-moment correlation test revealed a fair, statistically significant, negative correlation between normalized GM CSA and the number of back pain-related medical visits, (*r*(68) = -0.270; *P*<0.05).

**Table 3:** Gluteus maximus cross-sectional area measurements in patients with and without chronic low back pain.

Variables	Low Back Pain Patients (n = 36)		Control Patients (n = 32)		*P (95% CI)
	Mean (SD)	Range	Mean (SD)	Range	
Gluteus Maximus CSA- Right (cm^2^)	45.12 (10.10)	26.35–75.07	51.89 (12.54)	29.00–90.25	**0.016**
Gluteus Maximus CSA- Left (cm^2^)	44.67 (9.58)	27.68–71.07	51.51 (12.12)	28.29–83.94	**0.012**
Gluteus Maximus CSA- Combined (cm^2^)	89.79 (19.42)	54.03–146.14	103.39 (24.30)	57.29–174.19	**0.013**
Normalized Combined Gluteus Maximus CSA (CSA/Ht(cm))^2^	0.00328 (7.11E-4)	0.00205–0.00553	0.00377 (9.25E-4)	0.00217–0.00659	**0.017**

* Student's t-test; CSA = cross-sectional area, Ht = height, CI = confidence interval.

Statistical Significance (P < .05).

## Discussion

Our results show that the CSA of GM does vary significantly with chronic LBP prevalence in this sample of women between the ages of 40 and 69. There was also a fair correlation between GM CSA and the number of back pain-related medical visits in this sample [48]. A possible explanation for the GM atrophy seen in the present sample may be related to GM’s role in the lifting of loads. GM is such an important factor in lifting that it is believed to have played a crucial part in the development of bipedalism in humans, allowing the use of upper extremities in functional activities unavailable to quadrupeds, such as lifting, clubbing, and gathering [49]. The link between lifting and LBP has been well-established, especially with the lifting of excessively heavy loads and the use of improper body mechanics [24,38,39]. In particular, leaning forward while holding a load has been shown to increase the effective load on the lumbar IVDs substantially [23,25]. For example, Nachemson [23] found that holding a 20 kilogram load while bending forward only 20 degrees increased the lumbar IVD load by over 200 percent.

Gluteus maximus is functionally coupled with the paraspinal muscles in the performance of lifting from full flexion [27]. When arising from full trunk flexion into extension, most movement happens at the hip joint and is accomplished by GM and the hamstrings during the first 50% of the movement cycle. By 75% of extension, contributions from hip extensors and ES are about equal; and in the last 25% of extension to neutral, paraspinal muscles predominate [20–22]. During lifting of asymmetric loads, significant contralateral activation of the MF and ES muscles and significant ipsilateral activation of GM occurs [50]. Gluteus maximus is more active during a wide stance lift, but only with heavier weights [51]. Gluteus maximus also has important proximal attachments to the thoracolumbar fascia, and may play a direct role in spinal extension through this attachment [52].

Altered GM biomechanics have been noted in individuals with LBP. Kankaanpää et al [36] found that GM fatigued faster in a cohort of women with chronic LBP than in a group of healthy controls. Leinonen et al [22] found that both ES and GM were activated for substantially less time during both the flexion and extension cycle, and activated later in the cycle for trunk extension, in patients with LBP versus healthy controls. After 5 weeks of physical therapy, they found that the ES flexion/extension cycle activation time in patients with LBP was equal to that of the controls; GM, however, made no such recovery. Nadler et al [53] assessed right/left symmetry in GM strength in over 200 collegiate athletes, and correlated this with reported history of LBP and lower extremity pain. They found significant alterations in GM strength symmetry in females with a history of LBP.

Although the relationship between LBP, MF and other trunk muscles has been well-studied [15–19,54], we have found only two prior studies which have attempted to assess the relationship between LBP and gluteus maximus size. Skorupska et el [55] analyzed GM and other pelvic muscle volumes in 71 patients with LBP and leg pain and found a significantly smaller (p< 0.001) GM volume in their experiemental group compared with 29 healthy controls. On the other hand, Kamaz et al [56] found no significant differences in GM CSA among 36 sedentary women with chronic LBP compared with 34 control patients, with absolutely no trending towards significance (*P* = .503).

The association between GM CSA and LBP found in the present study provides a rationale for further research into GM’s role in LBP, the nature of its atrophy, and whether GM atrophy is premorbid or a result of LBP. With up to 90% of physical therapists using exercise as part of their treatment plan for patients with LBP, the present research also supports the use of therapeutic exercise interventions targeting GM for these patients [57]. The fact, however, that most exercises targeting GM also activate key trunk muscles such as MF and ES [58–60], will create a challenge when designing future studies to show the effects of isolated GM exercises on LBP.

### Limitations

Because we had limited access to patient medical history, other than reasons for visits and ICD-9 codes, it is possible that undisclosed, confounding medical diagnoses were present in both our control and chronic LBP samples. In addition, information on patient exercise habits or participation in physical therapy was not available and may have affected GM CSA. Gluteus maximus CSA measurements were performed by one reviewer who was not blinded to the medical histories of the patients, allowing potential introduction of bias into measurements. We utilized “back pain” as a “reason for visit” in our inclusionary criteria, and this may have inadvertently included patients with thoracic or cervical pain in our LBP sample. Finally, the atrophy seen in the present research may reflect incidental disuse atrophy seen with LBP, which is present in many muscle groups after prolonged immobilization or with a sedentary lifestyle [61]. Thus, any role in actual LBP causation due to GM atrophy cannot be extrapolated from the present research.

### Conclusions

Our research confirmed our hypothesis that GM atrophy would be greater in individuals with chronic LBP. Further research is indicated on GM CSA in individuals with varying age, sex, and LBP diagnoses. Research on the potential impact of exercise interventions targeting GM in individuals with chronic LBP is also indicated.
